# Design of Heavy Agricultural Machinery Rail Transport System and Dynamic Performance Research on Tracks in Hilly Regions of Southern China

**DOI:** 10.3390/s25144498

**Published:** 2025-07-19

**Authors:** Cheng Lin, Hao Chen, Jiawen Chen, Shaolong Gou, Yande Liu, Jun Hu

**Affiliations:** School of Mechatronics & Vehicle Engineering, East China Jiaotong University, Nanchang 330013, China; agr22900@163.com (C.L.);

**Keywords:** double-track rail transportation system, stress–strain analysis, modal analysis, eigensystem realization algorithm

## Abstract

To address the limitations of conventional single-track rail systems in challenging hilly and mountainous terrains, which are ill-suited for transporting heavy agricultural machinery, there is a critical need to develop a specialized the double-track rail transportation system optimized for orchard equipment. Recognizing this requirement, our research team designed and implemented a double-track rail transportation system. In this innovative system, the rail functions as the pivotal component, with its structural properties significantly impacting the machine’s overall stability and operational performance. In this study, resistance strain gauges were employed to analyze the stress–strain distribution of the track under a full load of 750 kg, a critical factor in the system’s design. To further investigate the structural performance of the double-track rail, the impact hammer method was utilized in conjunction with triaxial acceleration sensors to conduct experimental modal analysis (EMA) under actual support conditions. By integrating the Eigensystem Realization Algorithm (ERA), the first 20 natural modes and their corresponding parameters were successfully identified with high precision. A comparative analysis between finite element simulation results and experimental measurements was performed, revealing the double-track rail’s inherent vibration characteristics under constrained modal conditions versus actual boundary constraints. These valuable findings serve as a theoretical foundation for the dynamic optimization of rail structures and the mitigation of resonance issues. The advancement of hilly and mountainous rail transportation systems holds significant promise for enhancing productivity and transportation efficiency in agricultural operations.

## 1. Introduction

In southern China, hilly terrains are widely distributed, with orchards mostly situated on mountainous lands. The topography of this region is relatively complex [1]. Both fruit transportation and material handling in these regions require significant labor input. Traditional transportation methods are not only labor-intensive and inefficient but also costly due to terrain constraints. Following the implementation of agricultural machinery purchase subsidy policies, China’s agricultural machinery market experienced a decade of golden development, leading to unprecedented growth in the industry [2]. Large-scale agricultural equipment is increasingly being applied, yet transportation of such equipment remains a major challenge in hilly and mountainous regions due to topographical and traffic limitations.

The current single-track rail systems in hilly areas primarily serve the transportation of lightweight agricultural materials, with limited load capacities, thus failing to meet the transportation demands of large agricultural machinery. To address the difficulties in harvesting and heavy-load transportation in steeply sloped orchards, domestic scholars have conducted extensive research. Notable examples include the self-propelled dual-track orchard transporter designed by Xing [3] for steep slopes and the modular detachable single-traction dual-track transporter developed by Ouyang et al. [4] for mountainous orchards. Additionally, Ouyang et al. [5] designed a PLC-based automatic stopping system for dual-track transporters in mountainous orchards. This system employs hardware such as rotary encoders to enable operation within designated intervals, automatic round-trip functions, and precise positioning stops, enhancing the transporter’s safety, convenience, flexibility, and reliability. The development of dual-track transporters for mountainous orchards has significantly improved the production efficiency.

Double-track railways serve as a crucial component of railway transportation systems in mountainous orchards or other complex terrains. They directly support the smooth operation of transport vehicles and bear various dynamic loads from the vehicles and cargo. The structural performance of the rail is primarily reflected in their stress–strain behavior and vibration characteristics, which are closely related to the overall performance of the rail and the stability of the transportation system. Among these factors, the structural dimensions of the rail, such as wall thickness and support spacing, significantly influence their performance. A thinner wall and larger support spacing result in weaker resistance to deformation and make the stress–strain distribution more susceptible to changes. Due to the potential surface defects on the tracks (such as uneven welds, rust, etc.), variations in track gradient, differences in turning radii, as well as vehicle operations, such as acceleration, deceleration, and braking during transportation, double-track railways undergo complex load variations during operation. These load variations, coupled with factors such as the interaction between the running wheels and the tracks, as well as the elastic deformation of the track material itself, collectively result in continuous changes to the stress–strain state of the double-track railways, subsequently triggering vibration and noise issues. Therefore, conducting an in-depth analysis of the stress–strain and vibration characteristics of the tracks is of great significance for comprehensively evaluating the structural performance of the tracks and ensuring the stable and safe operation of the transportation system.

Finite element analysis (FEA) and experimental modal analysis (EMA) are effective methods for evaluating rail structural performance. Chen et al. [6] studied the modal parameters of sprayer boom trusses through modal experiments and FEA, employing a Multi-Objective Genetic Algorithm (MOGA) for parameter optimization, reducing maximum relative errors to 4.6%. Gharaghani et al. [7] combined FEA and modal testing to detect orange maturity, achieving at least 91% accuracy—an economical, effective, and reliable method for fruit maturity assessment. Saidin et al. [8] validated and updated the numerical FEA models of bridge structures using sensitivity-based updating methods. Comparing the experimental modal results, the differences in the first five dominant natural frequencies were less than 5%. Ji et al. [9] developed a mechanical deformation prediction system based on Effective Independence Modal Analysis, utilizing FEA and the Lanczos method to determine the first natural frequency of a transplanting machine as 102.68 Hz. This verified the accuracy of the modal analysis approach, providing references for subsequent vibration-reduction optimizations in transplanting machine arm structures and dimensions. The research also delved into the impacts of parameters, including wall thickness and support spacing, on the natural frequency of the rail, thereby laying a theoretical foundation for the subsequent optimization of the rail structure.

This paper presents an integrated structural design for a hilly and mountainous rail transportation system. Rail performance directly impacting transporter safety necessitates focused analysis and optimization of rail behavior. Utilizing modal analysis theory, a double-track rail finite element analysis (FEA) model was established through ANSYS Workbench. By integrating impact hammer experimental modal analysis (EMA) with the Eigensystem Realization Algorithm (ERA), dynamic characteristics of double-track rail under actual operating conditions were investigated. This approach yielded critical modal parameters, including natural modes, damping ratios, and mode shapes under realistic boundary constraints. The simulation results were compared with experimental measurements. Additionally, stress–strain experiments were conducted on rails under various loads to test the overall strain and analyze the stress variation processes, optimizing rail structures.

## 2. Design of Hilly and Mountainous Rail Transportation System

### 2.1. Overall Structure and Working Principle

**Figure 1:**
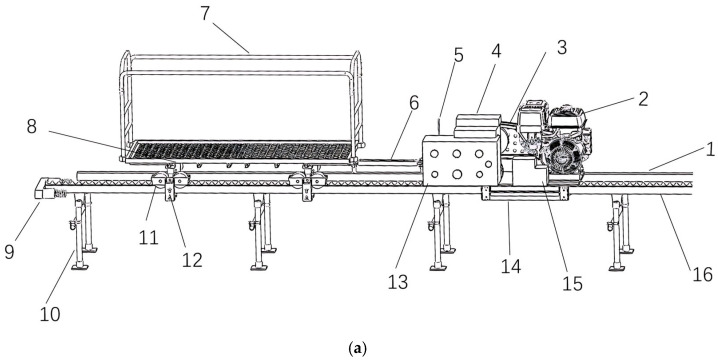

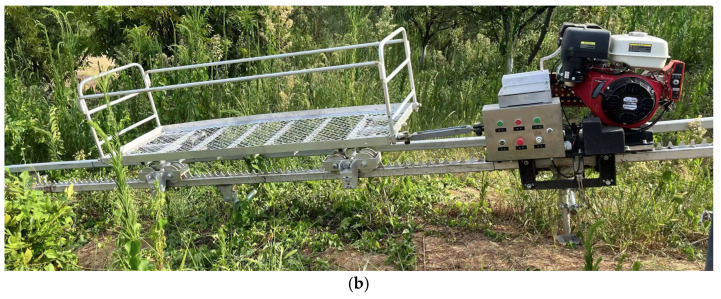
Design diagram of hilly and mountainous rail transportation system. (**a**). Three-dimensional diagram of hilly and mountainous rail transportation system components. 1. Auxiliary rail; 2. Gasoline engine; 3. Transmission mechanism; 4. Reduction gearbox; 5. RC receiver; 6. Coupling mechanism; 7. Trolley; 8. Load-bearing wheel assembly; 9. Position limiter; 10. Pile foundation; 11. Clamping wheel; 12. Anti-rollover wheel; 13. Main control cabinet; 14. Structural frame; 15. Storage battery; 16. Drive rail. (**b**). Real object diagram of hilly and mountainous rail transportation system.

The hilly and mountainous rail transportation system primarily consists of a gasoline engine, a reduction gearbox, a battery, a transmission mechanism, a drive assembly, a braking system, a clamping device, an anti-rollover mechanism, and a control unit, with the structure illustrated in Figure 1a. The gasoline engine provides propulsion for the entire transporter, while the battery supplies power to the remote-control system. After the gasoline engine outputs power, it undergoes deceleration through the transmission mechanism (belt drive) before being transmitted to the drive assembly (drive wheels) via the reduction gearbox. The drive wheels then propel the transporter along the rail through a rack-and-pinion engagement mechanism. A controllable braking device is installed at the input end of the reducer. This device’s engagement or disengagement states are regulated by the control system (e.g., buttons, switches) to manage power delivery to the drive wheels and determine the direction of motion (forward or reverse). Key technical specifications are presented in Table 1.

### 2.2. Main Component Design

#### 2.2.1. Track Structural Design

Current mainstream rail transportation systems, both domestically and internationally, primarily adopt single-rail or dual-rail configurations. As early as 1966, Japan had already constructed a steep-slope-applicable railway transporter named “MoonRack”. Inspired by this, MCK Machinery Co., Ltd., in South Korea designed a new type of forest monorail transporter, which has been widely applied in mountainous orchards, forest farms, airport construction, and other areas [10]. Building on the foundation of overseas rail-based transporters, domestic experts and scholars have successively developed various rail-based transporters. In 2008, Professor Zhang Yanlin’s team from Huazhong Agricultural University developed China’s first monorail orchard transporter and conducted a successful trial run in Yichang City, Hubei Province [11]. In 2012, Li from Huazhong Agricultural University designed a self-propelled large-slope dual-track orchard transporter [12]. Each model presents distinct advantages and disadvantages. Single-rail systems demonstrate superior terrain adaptability, high load capacity, and simple installation, yet compromise on safety performance. In contrast, dual-rail systems excel in steep gradient climbing, operational stability, large carrying capacity, and rapid transit speed, albeit at relatively higher construction costs.

To synergize the merits of both configurations while mitigating their respective drawbacks, an innovative self-propelled dual-rail track system is proposed. This system integrates a drive rail featuring rack-and-pinion design for power transmission and an idler rail without tooth structures for load-bearing and guidance. The dual-rail configuration enhances overall system stability. Subsequent design phases will focus on developing drive components tailored to this track structure.

**Figure 2:**
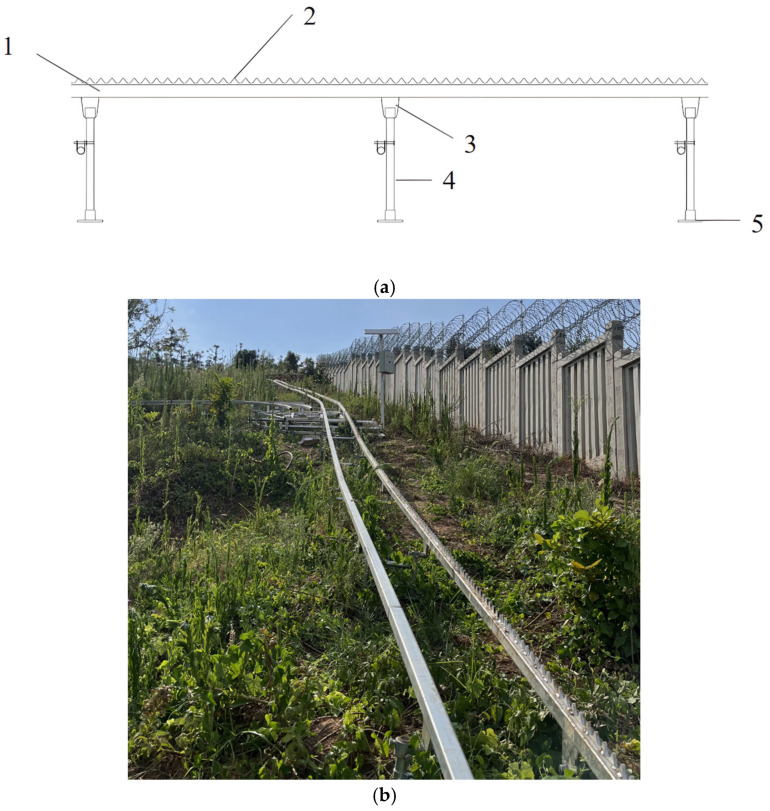
Design diagram of dual-rail track system. (**a**). Three-dimensional diagram of dual-rail track system. 1. Hollow square steel; 2. Rack teeth with chain-link profile; 3. Connecting plate; 4. Pile foundation system; 5. Support plate (**b**). Real object diagram of dual-rail track system.

To ensure compatibility with existing transportation infrastructure, the proposed system adopts a hollow square steel with outer dimensions of 50 × 50 mm and a wall thickness of 4 mm hollow square steel as the primary track structure. Rack segments are welded to the drive rail, with load-bearing wheels operating on both rails. The auxiliary rail primarily supports the transporter’s weight, while the drive rail’s tooth profile interfaces with the transporter’s drive mechanism to deliver propulsion. As illustrated in Figure 2, the structural assembly comprises hollow square steel beams, chain-type teeth, connecting plates, pile foundations, support plates, and welded joints for all component connections.

#### 2.2.2. Drive Components Design

**Figure 3:**
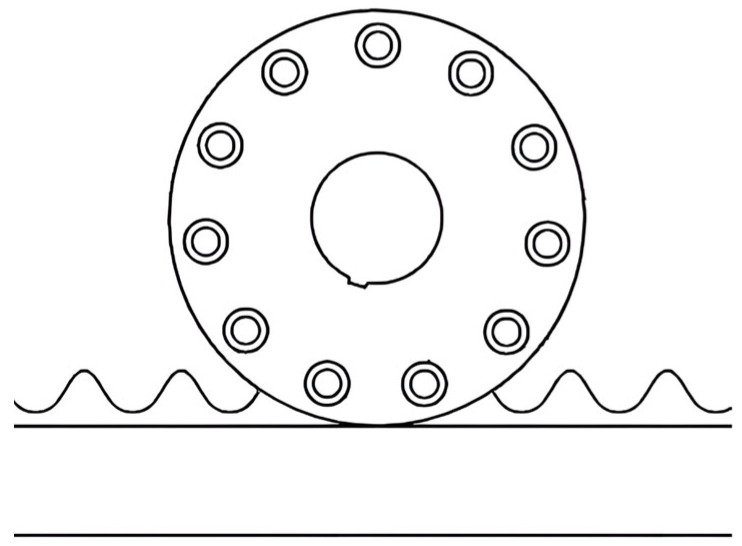
Drive mechanism transmission schematic.

The drive mechanism is designed based on the principle of dynamic traction. Employing gear and frictional force drive as the primary design system, sprocket and chain drive principles are utilized to develop specialized drive wheels and racks, substituting traditional gear transmissions. The transmission schematic is shown in Figure 3. When the drive wheel meshes with the sprocket-tooth rack, it produces minimal resistance torque with the smallest fluctuation amplitude. Under identical conditions, the sprocket-tooth rack demonstrates optimal integrated performance [13]. Based on this analysis, a 28A-type drive roller chain produced in China is selected for the drive design, with corresponding design parameters as follows.

(1) $$v=\frac{nD\pi }{60\times 1000}$$

(2) $$P=d\sin\frac{180{}^{\circ}}{z}$$

where *n*—Drive wheel rotational speed, r/min;

***v***—Drive wheel linear speed, m/s;

*D*—Drive wheel diameter, mm;

***P***—Chain pitch, mm;

*z*—Number of drive wheel pins;

***d***—Pin mounting diameter, mm.

According to the requirement of the linear speed control of drive wheels, where ***v*** ≥ 0.6 m/s, it can be derived that *D* ≥ 188.17 mm. This means that during the entire operation of the device, when the drive wheel diameter is 188.17 mm, the entire drive system of the device can be actuated.

#### 2.2.3. Track-Switching Mechanism Design

**Figure 4:**
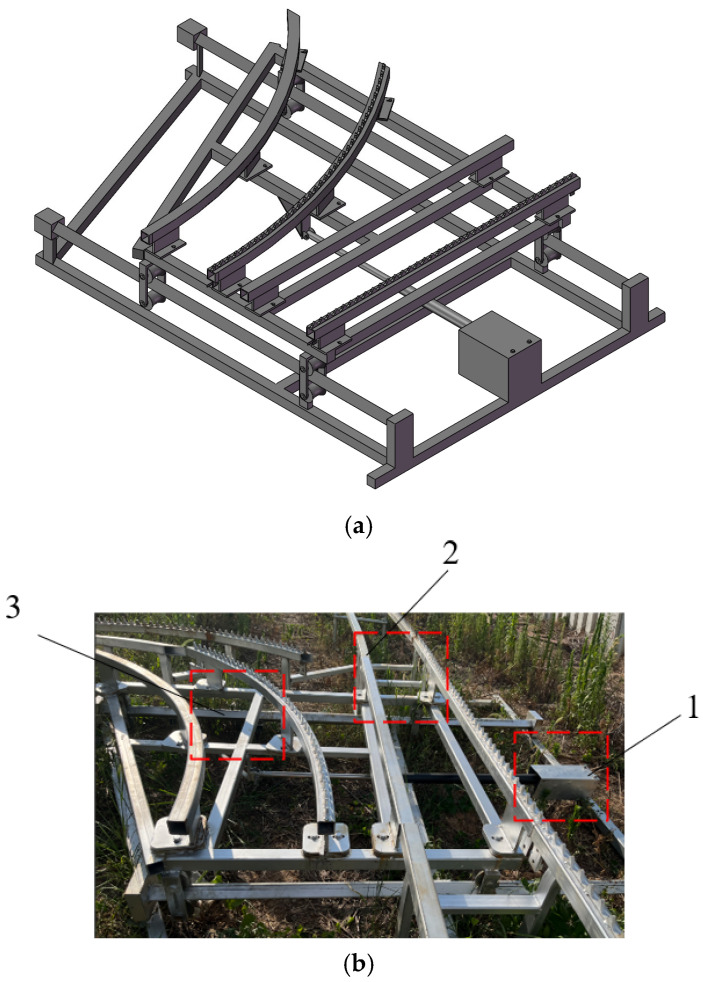
Design diagram of track-switching mechanism. (**a**). Three-dimensional diagram of track-switching mechanism. (**b**). Real object diagram of track-switching mechanism. 1. Telescopic motor; 2. Track 1; 3. Track 2.

To achieve a rapid and stable transfer of agricultural machinery and improve production and transportation efficiency, a track-switching mechanism is proposed here to transfer agricultural machinery to the required working platform. The track-switching device mainly consists of components such as a track system, a telescopic motor, a PLC controller, and a remote control. The track system, which includes track 1 and track 2, provides running paths for agricultural machinery. Figure 4a shows the SolidWorks modeling result, with the physical prototype presented in Figure 4b.

**Figure 5:**
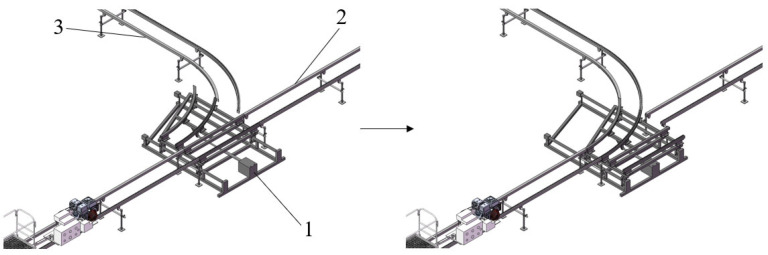
Schematic diagram of the track-switching process. 1. Track-switching mechanism; 2. Track 1; 3. Track 2.

Drawing inspiration from the railway track-switching principle, when the track-switching device needs to transfer agricultural machinery from track 1 to track 2, the operator issues a track-switching command using a remote control, and the signal is transmitted to the PLC controller. Upon receiving the command, the controller immediately sends an extension instruction to the telescopic motor. The telescopic motor extends and drives the connected track system to move, altering the track connection status to smoothly align track 1 with track 2, allowing the agricultural machinery to seamlessly enter track 2. After the machinery has completely passed through the track-switching area, the operator operates the remote control again to issue a reset command. The PLC controller then controls the retraction of the telescopic motor, and the track-switching mechanism returns to its initial position, ready for the next track-switching operation. The track-switching process is illustrated in Figure 5.

## 3. Experimental Equipment and Methodology

### 3.1. Experimental Equipment

The experimental instruments involved in this study include the INV3060V2 signal analysis system, INV9313 Impulse hammer, and INV9832-50 ICP Triaxial acceleration sensors, all developed by the China Orient Institute of Noise & Vibration. The resistance strain gauge used is model BE120-3AA-P500, with detailed parameters provided in Table 2.

### 3.2. Double-Track Rail Modal Analysis Experiment

#### 3.2.1. Modal Analysis Methods and Mathematical Model

Modal characteristics represent the inherent vibration attributes of a structural body, which depend solely on the dynamic characteristics of the structure itself rather than the applied external forces or excitation frequencies. Modal analysis is an analytical method based on vibration theory aimed at identifying modal parameters. Specifically, modal analysis utilizes the orthogonality of the natural modes of the system at various orders, employing a modal matrix composed of modal vectors at each order as a transformation matrix. This transforms the physical coordinates in the system’s vibration differential equations into modal coordinates, resulting in a set of independent, uncoupled motion equations described by modal coordinates and modal parameters. The essence of modal analysis is a coordinate transformation, where the transformation matrix is the modal matrix and each column vector represents a modal mode shape. The purpose of coordinate transformation is to decouple the equations, significantly reducing the number of equations and improving computational efficiency. This effectively lowers the difficulty of vibration analysis for large-scale complex structures. In addressing the dynamic characteristics of complex structures, theoretical modal analysis and experimental modal analysis constitute two complementary modern technical means, which have been widely applied in dynamic analysis across fields such as mechanical systems, aerospace engineering, building construction, and bridge engineering [14,15,16,17].

Generally, mechanical structures can be regarded as multi-degree-of-freedom linear vibration systems. The system motion differential equation for a double-track rail structure is given by

(3) $$M\{\ddot{x}\}+C\{\dot{x}\}+K\{x\}=F$$

where ***M*** is the mass matrix of the system; ***C*** is the damping matrix of the system; ***K*** is the stiffness matrix of the system; [6] is the displacement response vector at various points of the system; and ***F*** is the excitation force vector of the system.

When the system undergoes undamped free vibration, the excitation force ***F*** and damping matrix ***C*** are both zero. Substituting these conditions into Equation (3) gives

(4) $$M\{\ddot{x}\}+K\{x\}=F$$

Solving the linear differential Equation (4) yields

(5) $$\{x(t)\}=\{e^{j\omega t}\}\Phi$$

where ***Φ*** is the modal vector and *ω* is the natural modal frequency.

Substituting the solution of the homogeneous linear equation, Equation (5), into Equation (1) yields the following equation:

(6) $$(K-\omega^{2}M)\Phi \{e^{j\omega t}\}=0$$

A necessary and sufficient condition for a non-trivial solution to the above equation is

(7) $$\left|K-\omega^{2}M\right|=0$$

The natural frequency *ω_r_* and the corresponding natural mode shape *φ_r_* of the system can be solved using Equations (6) and (7). The modal vector can be expressed as

(8) $$\Phi =\{\varphi_{1}\varphi_{2}....\varphi_{n}\}$$

A vibration system typically possesses *n* natural vibration frequencies and their corresponding *n* natural mode shapes. Each such frequency and mode shape combination is equivalent to the vibration behavior of a single-degree-of-freedom system in a free state. Given that the nature of coordinate transformation is linear, the response of a linear system to any form of external excitation in the original physical coordinate system can be viewed as a linear superposition of the system’s modal vibrations. Therefore, modal analysis is also referred to as the modal superposition method [18].

#### 3.2.2. Principles of Finite Element Modal Analysis

**Figure 6:**
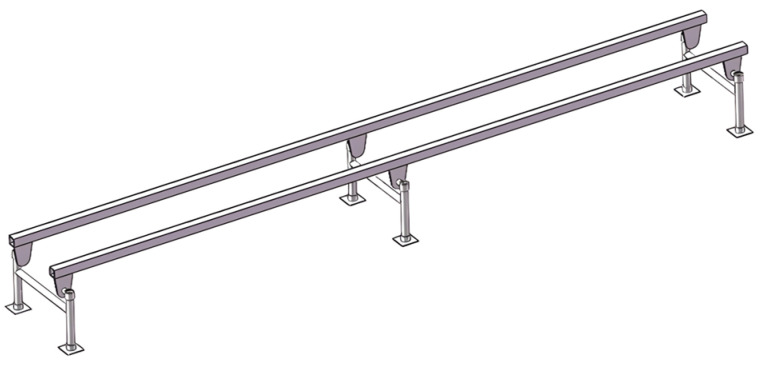
Simplified three-dimensional solid model.

**Figure 7:**
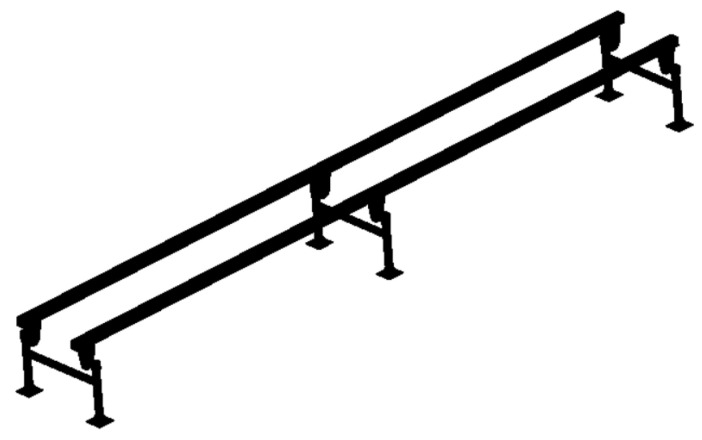
Finite element meshing model.

(1) Establishment of a three-dimensional model for the double-track rail: To streamline the analysis and enhance computational efficiency, structural features such as sprocket teeth, threaded holes, and mounting holes were omitted during the creation of the three-dimensional solid model for the double-track rail. Leveraging the seamless data transfer capability between the three-dimensional modeling software SolidWorks 2023 and ANSYS Workbench 2021 R1 Chinese Version, the three-dimensional solid model of the double-track rail was constructed using SolidWorks [19,20]. A simplified representation of the three-dimensional solid model for the double-track rail is presented in Figure 6, with a support spacing of 2050 mm, a track-to-track spacing of 400 mm, and a wall thickness of 4 mm.

(2) Preprocessing for modal analysis: Initially, a dual-rail track modal analysis project was established in the finite element analysis software ANSYS Workbench 2021 R1 Chinese Version. The three-dimensional solid model of the track, created in SolidWorks, was directly imported into the Geometry module to develop the finite element analysis model of the double-track rail. The Q235 material was assigned to the double-track rail in the created modal analysis project, with material parameters set as follows: elastic modulus of 201 GPa, Poisson’s ratio of 0.3, and density of 7860 kg/m^3^. Subsequently, the track finite element model was meshed in the Modal module, with an element size of 5 mm. The mesh was appropriately sized and uniformly distributed, resulting in a total of 1,252,470 nodes and 652,328 elements. The mesh generation results are shown in Figure 7.

After establishing a new coordinate system, the track was set to a 20° inclination angle using the part transformation options. Constraint conditions were applied to the double-track rail finite element model, with the pile base surface being fixed. Prior to performing the modal calculation, the modal solution options were configured in the analysis setting. To obtain the modal natural frequencies and mode shapes within a higher frequency range and facilitate a comprehensive analysis of the track structure modes from low to high order, the number of solution modes was set to 20.

#### 3.2.3. Experimental Modal Analysis

**Figure 8:**
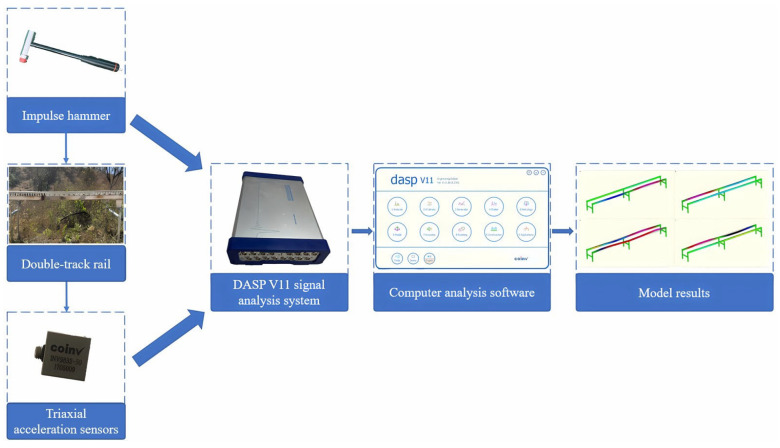
Flowchart of rail modal testing.

**Figure 9:**
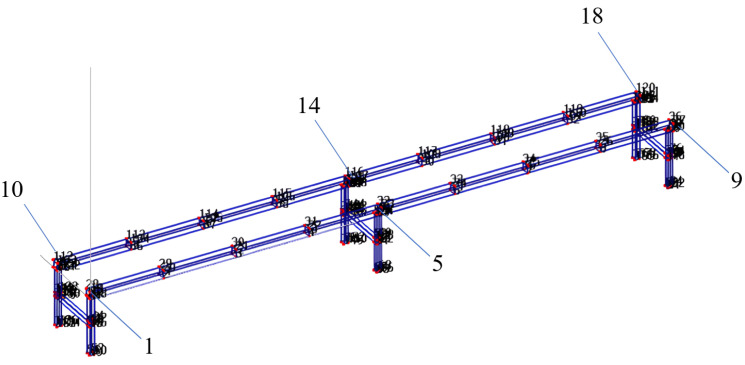
Experimental modal modeling.

**Figure 10:**
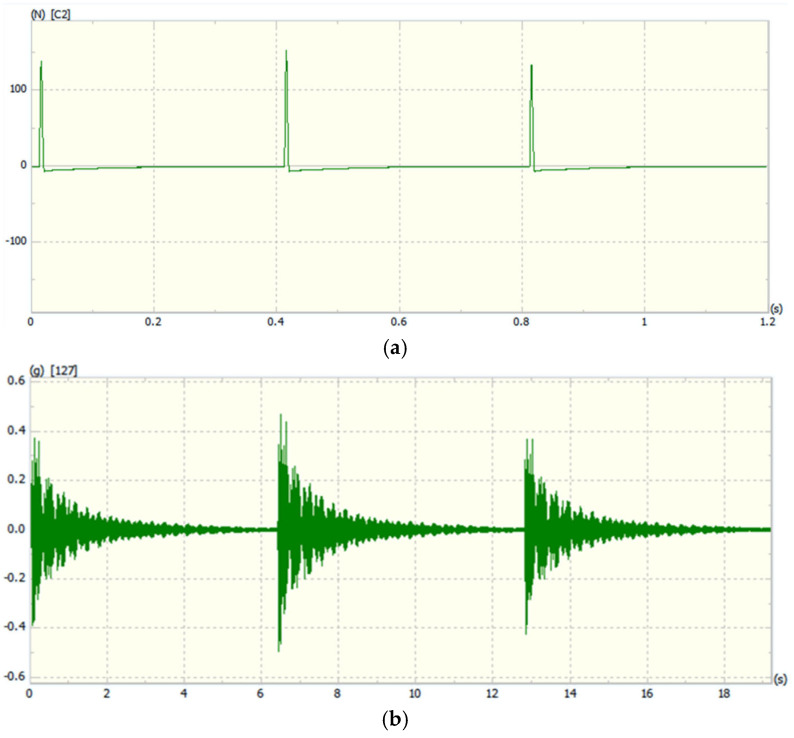
Single excitation and response signals. (**a**)**.** Single excitation signals. (**b**)**.** Response signals.

(1) Experimental methods and equipment: The modal testing experiment primarily employed the impulse hammer excitation method [21]. Appropriate excitation points were selected, and the track was tapped for measurement. The inherent modal parameters of the track were obtained by measuring the frequency response function [22]. The modal testing system mainly consisted of an impulse hammer, triaxial acceleration sensors, a signal analysis system, a computer, and data analysis software Dasp V11. The flowchart is shown in Figure 8. The triaxial acceleration sensors and the impulse hammer were connected to the channel sockets of the signal analysis system via cables. The data acquisition analyzer outputted the acquired excitation signals and time-domain signals to the computer data analysis software Dasp V11.

(2) Arrangement of measurement points: To enhance the accuracy of modal parameter identification, it is essential to arrange the impulse hammer tapping points reasonably and minimize the loss of modes. When selecting the excitation points, it is crucial to avoid the nodes of any structural mode shape to ensure that the acquired response signals have a high signal-to-noise ratio, prevent missing modes, and select excitation points with high stiffness to facilitate the transmission of excitation energy. The arrangement of measurement points on the track is shown in Figure 9. Two sections of the double-track rail were selected, and each section was divided into eight equal parts. The points were numbered from left to right as 1–9 and 10–18. Among these, points 1, 5, 9, 10, 14, and 18 are support points. The outermost support points of the two track sections—specifically points 1, 9, 10, and 18—were neglected here. The actual total number of measurement points was 14.

(3) Measurement of frequency response function: The experimental modal analysis method involves artificially applying certain excitations to the structure or directly utilizing excitations under natural conditions. By experimentally measuring the frequency response function, the transfer function of a multi-degree-of-freedom system is calculated. Subsequently, modal parameters characterizing the dynamic characteristics of the structure are obtained through curve fitting. The dynamic characteristics of the structure are mainly described by natural frequency, damping ratio, and mode shape [23]. To obtain high-quality modal parameters, it is necessary to ensure the accuracy of frequency response function measurement, the correctness of experimental setup, and the reasonableness of data analysis methods. The measurement method of the track frequency response function was conducted under the actual working conditions of the track, using impulse hammer excitation. To reduce experimental measurement errors, a multi-input multi-output (MIMO) testing method was employed [24]. This method allows for uniform energy distribution across measurement points, prevents insufficient energy at a single excitation point from resulting in insufficient signal-to-noise ratios at some response points, and more effectively distinguishes closely spaced modes. It also reduces the possibility of the reference point being located at a node, thereby improving the quality of the experiment. Five triaxial sensors were sequentially arranged at the measurement points, and points 2, 6, 13, and 15 were selected as excitation points. Each excitation point was tapped in two directions, with the excitation order being vertical (Y) first, followed by horizontal (Z). Each direction was excited three times to ensure there were no abnormal phenomena such as double taps or lack of excitation. Vibration signals were collected at each point. After collection, the sensors were moved to the second batch of measurement points, and four reference points were sequentially tapped. This process was repeated until data collection was completed for all measurement points. The sampling frequency of the response points was 640 Hz, and the sampling frequency of the excitation points was 1024 Hz. The single excitation and response point sampling results are shown in Figure 10. The sampling time for three excitations of a single degree of freedom at a single measurement point was approximately 19 s, and each excitation was sufficiently attenuated.

(4) Modal parameter identification: In this experiment, the Eigensystem Realization Algorithm (ERA) was employed to identify the natural frequencies of the track structure. This algorithm can accurately identify the modal parameters of the system and has advantages such as high computational efficiency and good stability. The basic principle involves constructing a Hankel matrix and performing singular value decomposition to extract modal parameters [25], as detailed below.

1. Hankel matrix construction:

The measurement data is *y*(*k*), and the Hankel matrix *H* is constructed as follows:

(9) $$H=\left(\begin{array}{cccc} y(0) & y(1) & \ldots & y(n-1)\\ y(1) & y(2) & \ldots & y(n)\\ \vdots & \vdots & \ddots & \vdots \\ y(m-1) & y(m) & \ldots & y(m+n-2) \end{array}\right)$$

where *n* is the number of rows and *m* is the number of columns of the Hankel matrix.

2. Singular value decomposition (SVD):

Perform SVD on the Hankel matrix, which can be decomposed as

(10) $$H=U{\sum V}^{T}$$

where U and *V* are orthogonal matrices, and Σ is a diagonal matrix containing singular values. The magnitude of the singular values reflects the importance of the system’s dynamic characteristics, with larger singular values corresponding to the system’s main modes.

3. Minimal realization extraction:

By truncating the results of the singular value decomposition and retaining the first r singular values (where r is the system order), the minimal realization of the system is obtained:

(11) $$\dot{x}(t)=Ax(t)+Bu(t)$$

(12) y(t)=Cx(t)+Du(t)

where *x*(*t*) is the state vector, *u*(*t*) is the input vector, *y(t)* is the output vector, *A* is the state matrix, *B* is the input matrix, *C* is the output matrix, and *D* is the direct transmission matrix.

4. Modal parameter calculation:

The natural frequency and damping ratio are determined by Equations (13) and (14), respectively.

(13) ωi=Re(λi)2+Im(λi)2

(14) ζi=−Re(λi)Re(λi)2+Im(λi)2

### 3.3. Dual-Track Stress–Strain Experiment

**Figure 11:**
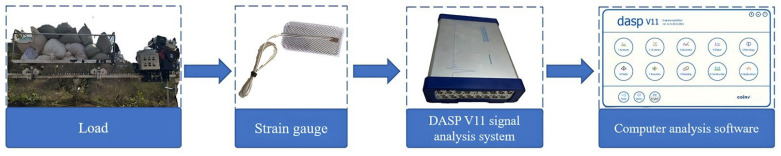
Stress–strain experiment flowchart.

A section of relatively smooth track surface was selected, and the bottom surface of the middle part of both tracks was polished with sandpaper. After spraying alcohol and wiping the polished track surface clean with cotton balls, electrical resistance strain gauges were glued to the polished areas of the track. The experimental procedure is shown in Figure 11. The strain gauges were connected to the channel sockets of the data acquisition and analysis instrument via cables, which transmitted the collected strain signals and time-domain signals to the computer data analysis software (Dasp V11). Check if the strain gauge is firmly attached, turn on the instrument, and simultaneously configure the relevant parameters: set the engineering unit to strain (µε), the calibration value to 0.001 mV/EU, and the gain multiplier to 10, and each wire was connected in sequence. Finally, the acquisition instrument was connected to the power supply. Bags of sand weighing 50 kg each were loaded onto the load carrier; the load carrier was loaded from 0 kg (empty) to 750 kg (full load), with a gradient of 150 kg for each set of tests for both upward and downward travel. Each set of tests was conducted twice, and data from the test points were recorded.

## 4. Results and Analysis

### 4.1. Modal Analysis Results

(1) Finite element analysis results: Using the solver in the Mechanical module of ANSYS Workbench 2021 R1 Chinese Version software, the natural frequencies and natural mode shapes of the first 20 orders of the dual-track were obtained. The natural frequencies and mode shape descriptions of each order of the track are shown in Table 3.

**Figure 12:**
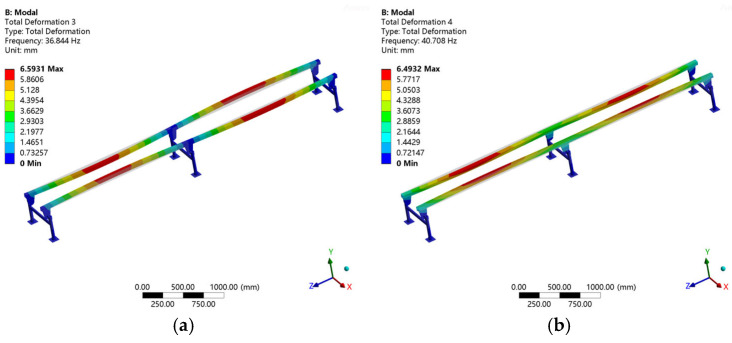

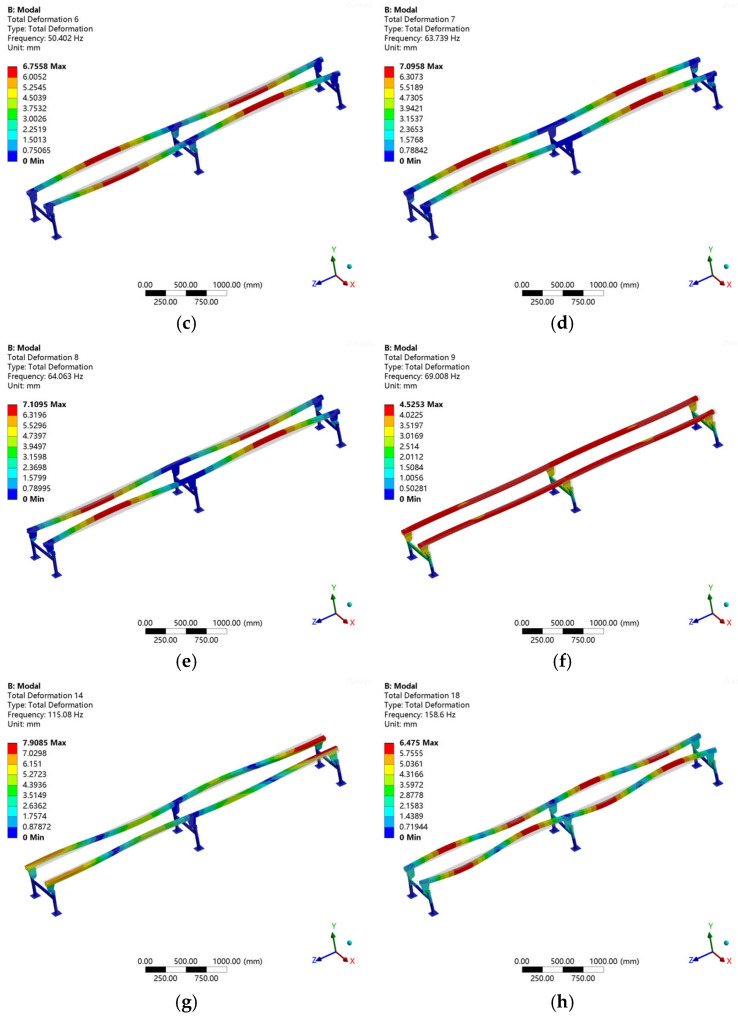
Partial vibration mode: (**a**). 3rd-order mode; (**b**). 4th-order mode; (**c**). 6th-order mode; (**d**). 7th-order mode; (**e**). 8th-order mode; (**f**). 9th-order mode; (**g**). 14th-order mode; (**h**). 18th-order mode.

To facilitate a comparison with the subsequent experimental modal results, only the 3rd, 4th, 6th, 7th, 8th, 9th, 14th, and 18th modal shapes are presented here. For detailed information, please refer to Figure 12.

(2) Experimental modal analysis results: Through the calculations of the frequency response functions at various measurement points on the rail and the identification of modal parameters, the natural frequencies, damping ratios, bandwidths, and corresponding natural mode shapes of 20 modal orders within the frequency range of 34.694 Hz to 197.991 Hz were obtained (see Table 4). The analysis of modal shapes revealed that the 1st and 2nd modal orders primarily exhibit first-order bending deflection in the Y-direction of the rail; the 3rd, 4th, 5th, and 9th modal orders show first-order bending deflection in the X-direction on one side of the rail; the 6th and 8th modal orders correspond to first-order torsional deflection in the XOY plane; and from the 10th modal order onward, second-order torsional deflection in the XOY plane becomes dominant. The first 9 modal orders are mainly characterized by first-order bending (primary bending) of the rail, while the subsequent 11 modal orders exhibit second-order bending (secondary bending).

The above 20 orders of modal analysis results were verified using the modal assurance criterion (***MAC***) [26] to compare the mode shapes of different orders and measure the correlation between each order. The modal assurance criterion between two modal vectors *Ψ_r_* and *Ψ_s_* is defined as follows:

(15) $$\mathrm{MAC}(\Psi r,\Psi s)=\frac{{\left|\left\{\Psi r^{*T}\Psi s\right\}\right|}^{2}}{\left(\Psi r^{*T}\Psi r)(\Psi s^{*T}\Psi s\right)}$$

**Figure 13:**
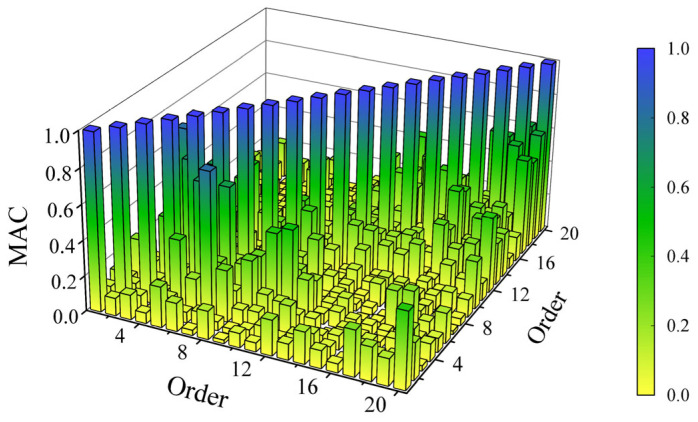
Modal assurance criterion analysis.

If *Ψ_r_* and *Ψ_s_* are the estimates of the same physical mode shape, then the modal assurance criterion should be close to 1. If *Ψ_r_* and *Ψ_s_* are the estimates of different physical mode shapes, then the modal assurance criterion should be very low [27]. The modal assurance criterion is shown in Figure 13. As can be seen from the figure, the diagonal of the modal assurance criterion is dominant, indicating that the experimental results are credible.

The modal participation factor is used to study the relative importance of each mode and the effectiveness of the selected input degrees of freedom. The participation factor ***MP****_nr_* of input *n* for mode r is calculated using the following formula:

(16) $${\mathrm{MP}}_{nr}=\sum_{x=1}^{N0}\left|A_{xnr}\right|$$

where *x* represents the output degree of freedom; *A_xnr_* represents the residue of output *x*, input *n*, and mode *r*.

**Figure 14:**
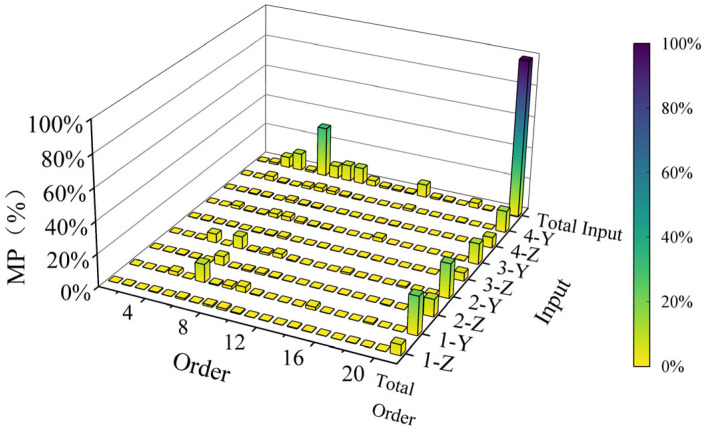
Modal participation factor analysis.

**Figure 15:**
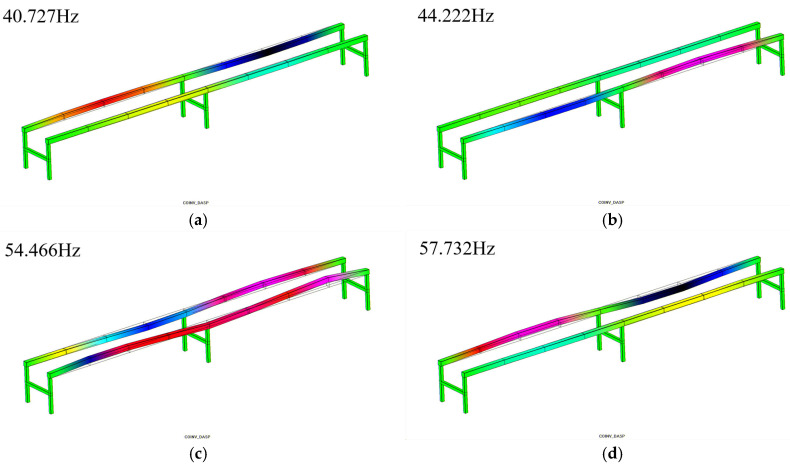

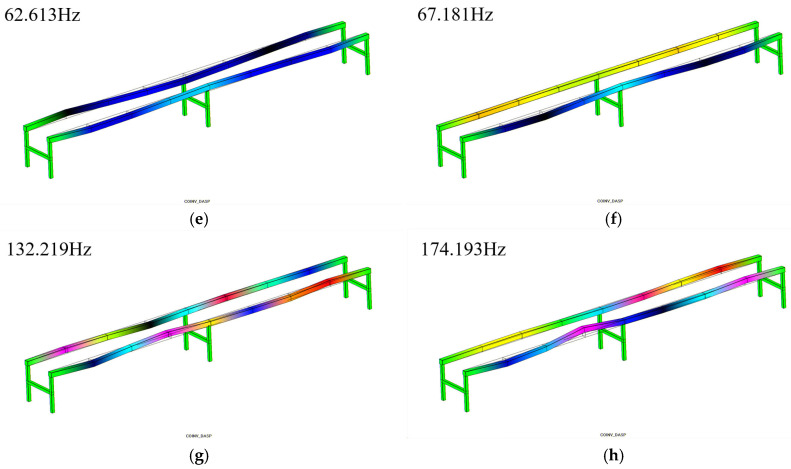
Partial track experimental modal mode shapes: (**a**). 3rd-order mode; (**b**). 4th-order mode; (**c**). 6th-order mode; (**d**). 7th-order mode; (**e**). 8th-order mode; (**f**). 9th-order mode; (**g**). 14th-order mode; (**h**). 18th-order mode.

Figure 14 shows the modal participation factors. It can be seen that the participation factors of the 3rd-, 4th-, 6th-, 7th-, 8th-, 9th-, 14th-, and 18th-order modes are 6.77%, 10.89%, 31.65%, 7.74%, 10.02%, 10.02%, 8.12%, and 3.21%, respectively, accounting for 88.42% of the total. Therefore, only their modal mode shapes are listed for reference. For details, please refer to Figure 15.

(3) At the same time, the theoretically calculated values were compared, and their errors were analyzed. The comparison between the experimental and calculated values of the overall modal frequencies of the first 20 orders is shown in Table 5.

A comparison was made between the experimental modal analysis results and the finite element analysis results, analyzing them in terms of frequency and mode shape.

1. Frequency analysis

The maximum error between the experimental and calculated values was 18% (15th order), and the minimum error was 0.176% (1st order). Among the modes that are relatively important to the whole, the clear majority of errors for the 3rd-, 4th-, 6th-, 7th-, 8th-, 9th-, 14th-, and 18th-order modes are within 10%.

2. Mode shape analysis

Comparing the mode shapes of the 3rd, 4th, 6th, 7th, 8th, 9th, 14th, and 18th orders, it was found that only the 9th-order theoretical mode shape differed from the experimental mode shape, while the remaining mode shapes were basically consistent.

The reasons for the differences between the two are as follows: The two analysis methods used different boundary conditions. The hammer impact method modal test was conducted under the actual working conditions of the track, which can more realistically reflect the actual boundary conditions of the track structure. However, in the finite element modal analysis presented in this paper, only the bottom surface of the pile foundation was fixed and supported, which cannot truly reflect the actual force conditions of the track. In addition, to improve the computational efficiency, the model was simplified during the finite element modal analysis of the track, ignoring some additional components. However, the actual working track includes additional masses such as screws and chamfers at the track joints, which are also important reasons for the differences in the results. This difference has a relatively minor impact on low-order modes but becomes more significant for high-order modes, thereby causing an increase in the error.

### 4.2. Stress–Strain Experiment Results

After completing the six groups of stress tests, the time-domain signal images and test data of the impact tests were saved for subsequent processing and analysis of the test stress signals.

**Figure 16:**
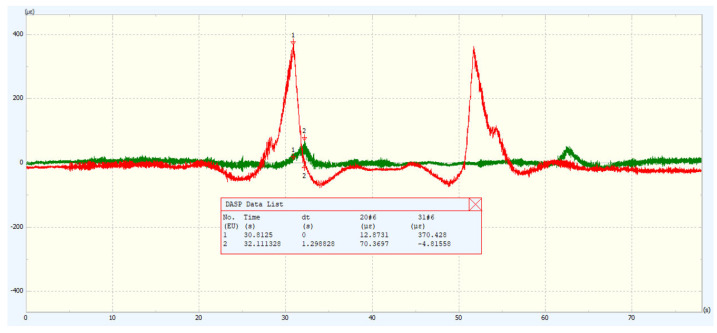
Auxiliary rail no-load and full-load stress–strain diagrams.

The data signal analysis software (Dasp V11) was used to process and analyze the test data. This included importing the data files into the software (Dasp V11), analyzing the time-domain signals, and finding the maximum stress values. To more clearly express the entire stress variation process, the typical working conditions of the load-carrying vehicle, no-load and full-load, were selected for explanation, as shown in Figure 16.

In order to more accurately evaluate the variation of rail strain with load, the average of the maximum strain values of the midpoint data from the upstream and downstream measurements was taken as the final strain value experienced by the rail. The statistical data is shown in Table 6.

When the load-carrying vehicle is no-load, the main load-bearing is on the drive rail. The reason for this is that the power head is on the drive rail. As the load increases, the stress variations of the auxiliary rail and the drive rail begin to approach each other. Moreover, when the load is relatively small, the stress and strain amplitude changes of the power rail are not significant. Under full load conditions, the maximum strain amplitude of the power rail is 468.89 µε, and the stress amplitude is 93.78 MPa. The maximum strain amplitude of the auxiliary rail is 391.46 µε, and the stress amplitude is 78.29 MPa. All these values are lower than the yield limit of the rail. Overall, as the load of the load-carrying vehicle increases, the strain amplitude experienced by the rail increases and varies greatly, presenting a positive correlation.

## 5. Analysis of the Influence of Structural Parameters on the Natural Frequency of the Rail

### 5.1. Wall Thickness

The three-dimensional solid models of double-track rail with wall thicknesses of 3 mm and 5 mm, respectively, were established. Then, these models were imported into Workbench for finite element modal analysis. The mesh generation method and modal solution option settings remain the same as those described previously. The natural modal frequencies of the double-track rail with the two different thicknesses are presented in Table 7 below.

It can be observed from Table 7 that the impact of wall thickness on the natural frequency of the rail is manifested as a decrease in the natural modal frequencies of corresponding orders with an increase in wall thickness.

### 5.2. Support Spacing

**Figure 17:**
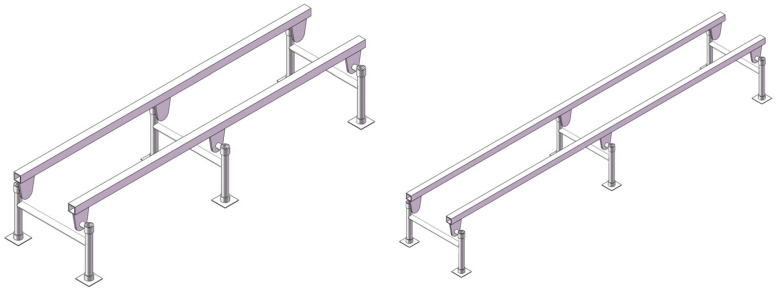
Three-dimensional solid models of tracks with support spacings of 1000 mm and 1500 mm.

The three-dimensional solid models of double-track rail with support spacings of 1000 mm and 1500 mm, respectively, were established. The three-dimensional solid models of double-track rail with a wall thickness of 4 mm and these two different support spacings are shown in Figure 17.

Through finite element modal analysis conducted in Workbench, the natural modal frequencies of the double-track rail with two different support spacings were obtained, as presented in Table 8. It can be seen from the table that as the support spacing increases, the corresponding modal natural frequencies decrease.

By considering the impacts of wall thickness and support spacing on the natural frequency of the rail, an analysis was conducted on the excitation frequency of the drive system. In this drive system, a gasoline engine serves as the power source, with the number of cylinders ***n*** = 1 and a rotational speed ***N*** = 3600 r/min. The combustion excitation frequency ***f_c_*** and the inertial force excitation frequency ***f_r_*** of reciprocating components are among the main sources of vibration in internal combustion engines [28,29], and their fundamental frequencies are closely related to the engine speed and the number of cylinders. The calculation formulas for the combustion excitation frequency ***f_c_*** and the inertial force excitation frequency ***f_r_*** of reciprocating components are as follows:

(17) fc=Nn120

(18) fr=N60

In the formula, ***N*** represents the rotational speed in r/min, and ***n*** represents the number of cylinders.

When the rotational speed is 3600 r/min, the combustion excitation frequency fc of this gasoline engine is 30 Hz, and the inertial force excitation frequency ***f_r_*** of the reciprocating components is 60 Hz. Therefore, it is possible to appropriately increase the natural frequency of the rail by reducing the wall thickness or the support spacing so as to avoid resonance.

## 6. Conclusions

This paper investigates the structural performance of the double-track rail for mountain orchard rail transporters through the overall design of a hilly and mountainous rail transportation system. By analyzing the stress–strain conditions of the rail and employing both the finite element modal analysis and experimental modal analysis methods, the structural performance of the double-track rail was studied. The main conclusions are as follows:

(1) The drive rail in the double-track rail bears most of the weight. However, under a certain load, the strain and stress amplitudes of the auxiliary rail and the drive rail gradually approach each other, and both rails share the load equally. This dual-rail track has a good load-bearing capacity. Furthermore, the strain conditions can provide a basis for the overall structural optimization of the hilly and mountainous rail transportation system.

(2) To study the natural vibration characteristics of the track structure, a modal analysis was performed on the finite element model of the double-track rail to obtain the theoretical modal calculation results. According to the obtained modal mode shapes, bending deformation of the rail is one of the main modes, and the maximum deformation usually occurs in the middle or near the end of the rail. Most deformations occur in the left–right (*X*-axis) direction. This analysis can be used to evaluate the rationality of the track structure design and provides a basis for structural improvement.

(3) Through the experimental modal analysis of the double-track rail under actual working conditions, the vibration characteristics of the track under real working conditions were obtained. It was found that the low-order modal vibration modes of the double-track rail are mainly dominated by bending deformation in a single direction, while the high-order modal vibration modes are mainly dominated by planar torsional deformation. The first 9 orders of rail deformation are mainly first-order bending, and the latter 11 orders are second-order bending.

(4) Combining the theoretical modal analysis results with the experimental modal analysis results, the operating frequency range of the drive system was analyzed. To avoid resonance issues, the natural frequency range of the double-track rail can be increased by installing reinforcing plates on the outer side of the track and reducing the track wall thickness or the ratio of support spacing. This provides a basis for the structural design and dynamic characteristic optimization of the double-track rail.

## Figures and Tables

**Table 1:** Main technical parameters of the system.

Parameter	Value
Weight	230 kg
Overall dimensions	3050 × 600 × 1020 mm^3^
Power	8.5 kW
Load capacity	≥750 kg
Travel speed	≥0.6 m/s
Maximum operating slope	≥40°
Minimum braking distance	≤10 cm
Remote control range	60 m

**Table 2:** Experimental instruments and key parameters.

Equipment	Model	Key Specifications
Signal analysis system	INV3060V2	Channels: 16; ADC resolution: 24-bit; Maximum sampling rate: 102.4 KHz/channel.
Impulse hammer	INV9313	Measurement range: 0~25,000 N; Sensitivity: 0.20 mV/N.
Triaxial acceleration sensors	INV9832-50	Sensitivity: 100 mV/g; Range: ±50 g; Nonlinearity: ≤1%; Frequency response: 1–10 kHz (±1 dB).
Strain gauge	BE120-3AA-P500	Resistance: 119.9 ± 0.1 Ω; Gauge factor: 2.17 ± 1%.

**Table 3:** Natural modal frequency of track under constraint conditions.

Order	Frequency/Hz	Order	Frequency/Hz
1	34.755	11	82.127
2	35.748	12	96.124
3	36.844	13	106.07
4	40.708	14	115.08
5	49.929	15	116.08
6	50.402	16	128.82
7	63.739	17	157.38
8	64.063	18	158.60
9	69.008	19	173.2
10	73.618	20	180.72

**Table 4:** Experimental modal analysis results.

Order	Frequency/ Hz	Damping Ratio/%	Mode Shape
1	34.694	2.625	1st-order bending deflection in Y-direction
2	38.125	0.409	1st-order bending deflection in Y-direction
3	40.727	0.607	1st-order bending deflection in X-direction (unilateral)
4	44.222	0.513	1st-order bending deflection in X-direction (unilateral)
5	50.184	1.077	1st-order bending deflection in X-direction (unilateral)
6	54.466	1.758	1st-order torsional deflection in XOY plane
7	57.732	1.083	1st-order bending deflection in X-direction (unilateral)
8	62.613	1.572	1st-order torsional deflection in XOY plane
9	67.181	0.774	1st-order bending deflection in X-direction (unilateral)
10	72.090	1.456	2nd-order torsional deflection in XOY plane
11	77.408	1.182	2nd-order torsional deflection in XOY plane
12	84.609	1.070	2nd-order torsional deflection in XOY plane
13	106.499	0.831	2nd-order bending deflection in Y-direction
14	132.219	1.110	2nd-order torsional deflection in XOY plane (X-dominant)
15	142.458	0.769	2nd-order torsional deflection in XOY plane
16	151.199	0.808	2nd-order torsional deflection in XOY plane
17	165.168	1.291	2nd-order torsional deflection in XOY plane (X-mode)
18	174.193	2.299	2nd-order torsional deflection in XOY plane
19	181.744	1.613	2nd-order torsional deflection in XOY plane (unilateral)
20	197.991	2.555	2nd-order torsional deflection in XOY plane

**Table 5:** Experimental vs. calculated modal frequencies.

Order	Experimental Value/Hz	Calculated Value/Hz	Error/%	Order	Experimental Value/Hz	Calculated Value/Hz	Error/%
1	34.694	34.755	0.176	11	77.408	82.127	6.095
2	38.125	35.748	6.235	12	84.609	96.124	13.610
3	40.727	36.844	9.534	13	106.499	106.070	0.393
4	44.222	40.708	7.946	14	132.219	115.080	12.963
5	50.184	49.929	0.510	15	142.458	116.080	18.516
6	54.466	50.402	7.463	16	151.199	128.820	14.801
7	57.732	63.739	10.405	17	165.168	157.380	4.715
8	62.613	64.063	2.314	18	174.193	158.600	8.957
9	67.181	69.008	2.720	19	181.744	173.200	4.701
10	72.090	73.618	2.118	20	197.991	180.72	8.723

**Table 6:** Final strain and stress value of the rail.

Load/kg	Strain/µε		Stress/MPa	
	Drive Rail	Auxiliary Rail	Drive Rail	Auxiliary Rail
0	331.52	68.56	66.30	13.71
150	333.22	193.85	66.64	38.77
300	338.61	282.40	67.72	56.48
450	348.82	310.80	69.76	62.16
600	387.66	323.26	77.53	64.65
750	468.89	391.46	93.78	78.29

**Table 7:** The natural modal frequencies of tracks with wall thicknesses of 3 mm and 5 mm.

Wall Thickness/mm	Order and Frequency/Hz						
	1	2	3	4	5	6	7
3	37.321	37.953	38.304	43.769	50.554	50.950	65.180
5	32.327	34.482	35.675	38.245	48.568	49.067	62.051
	8	9	10	11	12	13	14
3	65.458	72.794	77.638	87.015	101.530	111.490	120.790
5	62.387	65.553	69.974	78.176	91.943	101.740	110.540
	15	16	17	18	19	20	
3	120.840	135.240	160.300	161.410	176.660	184.530	
5	112.180	123.270	153.560	154.770	169.830	176.510	

**Table 8:** The modal frequencies of double-rail tracks with spaces of 1000 mm and 1500 mm.

Support Spacing/mm	Order and Frequency/Hz						
	1	2	3	4	5	6	7
1000	56.497	71.821	90.529	98.093	105.630	114.210	129.300
1500	44.465	54.919	58.661	63.680	80.624	84.840	88.397
	8	9	10	11	12	13	14
1000	176.280	177.710	180.090	206.270	221.990	223.810	240.390
1500	90.842	101.680	112.970	113.690	127.890	140.630	160.640
	15	16	17	18	19	20	
1000	280.550	371.700	372.100	408.210	422.280	474.750	
1500	183.840	192.720	254.620	270.690	271.990	289.410	

## Data Availability

The data presented in this study are available on request from the corresponding author.
